# Statistical colour models: an automated digital image analysis method for quantification of histological biomarkers

**DOI:** 10.1186/s12938-016-0161-6

**Published:** 2016-04-27

**Authors:** Jie Shu, G. E. Dolman, Jiang Duan, Guoping Qiu, Mohammad Ilyas

**Affiliations:** College of Computer Science and Technology, North China University of Technology, Beijing, China; Beijing Key Laboratory on Integration and Analysis of Large-scale Stream Data, Beijing, China; University of Nottingham, Nottingham, UK; School of Economic Information Engineering, Southwestern University of Finance and Economics, Chengdu, China; School of Computer Science, University of Nottingham Ningbo Chian (UNNC), Ningbo, China; Queens Medical Center NHS Trust (QMC), Nottingham, UK, Nottingham, UK

**Keywords:** Colour detection, Statistical model, Colour deconvolution, Digital pathology, Histological image processing, Biomarker quantification, Software

## Abstract

**Background:**

Colour is the most important feature used in quantitative immunohistochemistry (IHC) image analysis; IHC is used to provide information relating to aetiology and to confirm malignancy.

**Methods:**

Statistical modelling is a technique widely used for colour detection in computer vision. We have developed a statistical model of colour detection applicable to detection of stain colour in digital IHC images. Model was first trained by massive colour pixels collected semi-automatically. To speed up the training and detection processes, we removed luminance channel, Y channel of YCbCr colour space and chose 128 histogram bins which is the optimal number. A maximum likelihood classifier is used to classify pixels in digital slides into positively or negatively stained pixels automatically. The model-based tool was developed within ImageJ to quantify targets identified using IHC and histochemistry.

**Results:**

The purpose of evaluation was to compare the computer model with human evaluation. Several large datasets were prepared and obtained from human oesophageal cancer, colon cancer and liver cirrhosis with different colour stains. Experimental results have demonstrated the model-based tool achieves more accurate results than colour deconvolution and CMYK model in the detection of brown colour, and is comparable to colour deconvolution in the detection of pink colour. We have also demostrated the proposed model has little inter-dataset variations.

**Conclusions:**

A robust and effective statistical model is introduced in this paper. The model-based interactive tool in ImageJ, which can create a visual representation of the statistical model and detect a specified colour automatically, is easy to use and available freely at http://rsb.info.nih.gov/ij/plugins/ihc-toolbox/index.html. Testing to the tool by different users showed only minor inter-observer variations in results.

## Background

Histopathological assessment is a crucial clinical diagnostic technique. A wide range of immunohistochemical and histochemical stains are available to assist histological assessment by providing contrast between a protein (or cell type) of interest and background tissue. These stains colour the target antigens or proteins, called biomarkers, with different chromogens to visualise them to assist visual microscopic analysis [1].

Diaminobenzidene (DAB) is one of the most commonly used stains in immunohistochemistry (IHC); it stains a variety of biomarkers, such as P53 and elastin dark brown. P53 is a tumour suppressor protein expressed predominantly in cell nuclei. Inspecting the distribution of P53, which has been shown to be over expressed in malignant tumours, aids the diagnosis of colorectal cancer [2]. Elastin is the main component of elastic fibres, usually found in arterial walls. Elastin is also found in the liver and is present a higher density in liver fibrosis and cirrhosis, making it a potentially useful biomarker of the severity of liver fibrosis [3]. A counter-stain, the haematoxylin stain, which stains the background tissue blue, is normally used a fter DAB staining. Picro-Sirius Red (PSR) is a histochemical stain commonly used to detect fibrosis in liver biopsies [4]. The connective tissue matrix is stained red by PSR whilst background liver tissue appears a pale yellow colour.

**Fig. 1:**
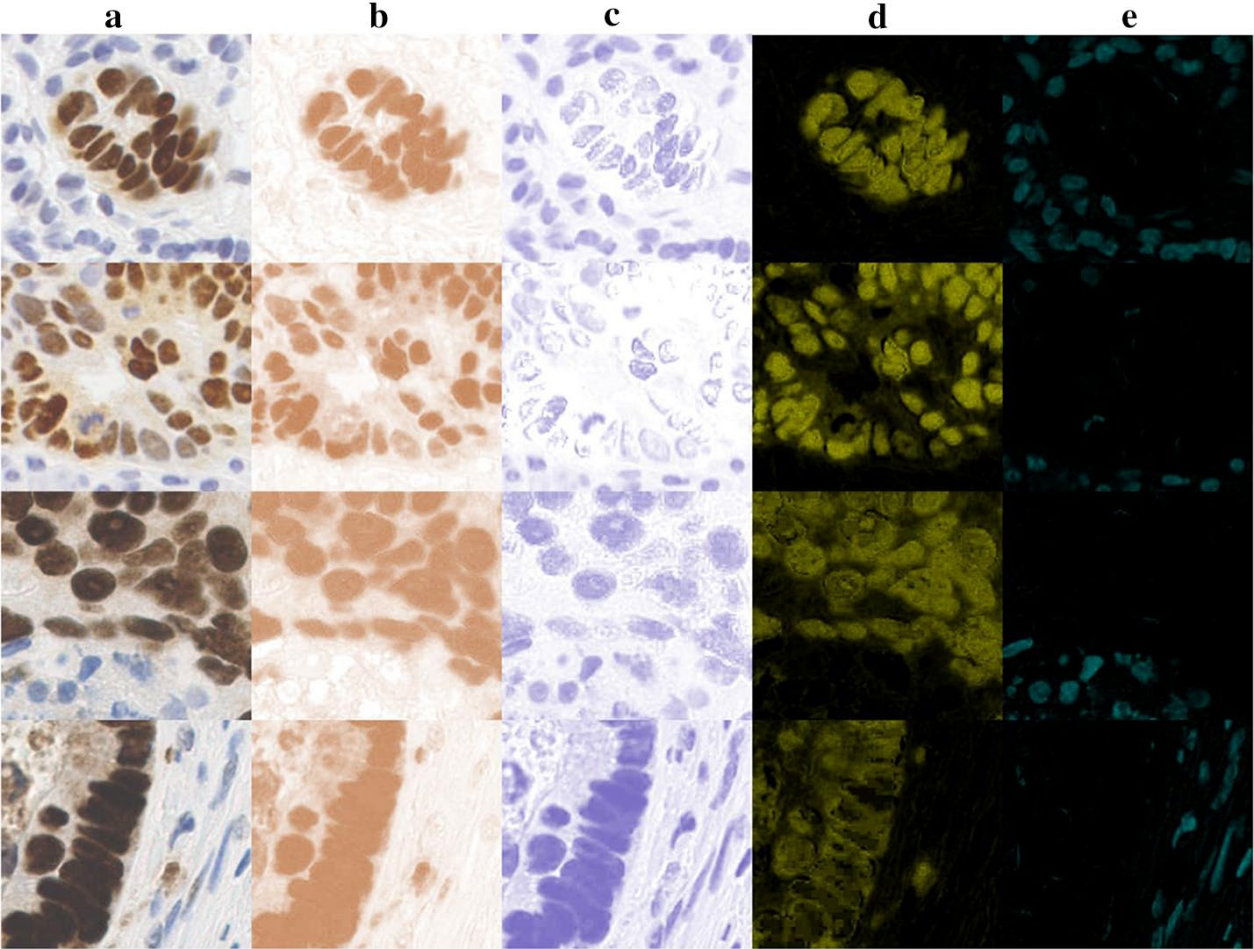
The previous methods detected stain colour on DAB stained samples. **a** Column is the original DAB stained samples, the upper two samples from WS images and lower two samples from TMA images; **b**, **c** columns are the colour deconvolution [10] detected *brown* and *blue colour*; **d**, **e** columns are the CMYK model [9] detected *brown* and *blue colour* in Y and C channel

Many methods have been used to quantify the stain colour in IHC images [5–7]. However, misdetection is a common problem when two or more chromogens with overlapping absorption spectra are used on one slide [7, 8]. For example, the brown colour pixels were missing from a dark DAB-stained area when the single Y channel in the CMYK model was used for classification [9] (see Fig. 1d). Colour deconvolution (CD) [10], one of the most popular methods, falsely recognized the brown colour pixels in the dark DAB-stained area as blue colour pixels (see Fig. 1b, c). Colour deconvolution exploits differences in the light absorption spectra of different colour stains, but because it is based on a linear light absorption algorithm detection accuracy may be reduced if the light is not linearly absorbed by the stain, as is the case with DAB stain [11].

In this study we treated the detection of stained pixels as a colour detection problem in computer vision. Pixels stained a specified colour, positive colour pixels, are considered as a group of pixels which can be extracted from the background, the negative colour pixels. The method of stain colour detection in digital IHC images proposed here is a statistical colour detection model. A model is created from a huge collection of colour pixels that contains both the positive and negative colour pixels in the image. A maximum likelihood classifier based on statistical models of the positive and negative pixels, is used to classify pixels in digital slides into positively and negatively stained pixels automatically.

We constructed the model [6] and have demostrated it has high accuracy in detection of DAB stain in colon cancer and PSR stain in liver cirrhosis [5, 7]. In this paper, we first replenished previous experiments by training to get corresponding CD vectors rather than only using built-in vector. Previous works only presented the evaluation of corresponding models, which were trained by corresponding images or mixed images, to colour detection result in each dataset. Thus, we then added many tests to evaluate the variations of model detecting results across datasets. And finally we compared the detection result variations between model, trained by images all from another dataset, and corresponding model. The paper is organized as follows:

- "Methods" section describes how we developed the statistical colour model for stain colour detection.
- "Softwares" section introduces the interactive tool built in ImageJ.
- "Experiments and discussion" section describes tests of the model-based tool in several datasets.

The results of all these tests demonstrated the robustness and effectiveness of this statistical colour detection tool.

## Methods

### Statistical model

The statistical model presented in [6, 12] has been used for the detection of positive immunostain colour. For model construction, the labelled colour pixels are arranged into a colour histogram in a specific colour space. For example, in RGB colour space, the pixels are quantized into RGB colour bins. The probability of positive or negative for each bin is separately calculated as follows.

1 $$\begin{aligned} Prob(RGB|S)=\frac{\#S[RGB]}{N_{S}}\quad Prob(RGB|\overline{S})=\frac{\#\overline{S}[RGB]}{N_{\overline{S}}} \end{aligned}$$

In (Eq. 1) *S* is the positively stained class and $\overline{S}$ is the negatively stained class, $\#S[RGB]$ the number of positively stained pixels with a colour value of [*RGB*], $\#\overline{S}[RGB]$ the number of negatively stained pixels with a colour value of [*RGB*], $N_{S}$the total number of positively stained pixels and $N_{\overline{S}}$ the total number of negatively stained pixels.

### Maximum likelihood

The classification of this bin belongs to the target stain or the background is determined by the maximum likelihood ratio approach.

2 $$\begin{aligned} \frac{Prob(RGB|S)}{Prob(RGB|\overline{S})} \ge \theta \quad 0 \le \theta \le 1 \end{aligned}$$

The value of $\theta$ is obtained through the experimental results presented in "Experiments and discussion" section.

### Colour models

It is recognised that Red, Green and Blue (*RGB*) colour space is not suitable for image analysis. One reason for this is that chromaticity information and brightness (luminance) information are mixed together in this colour space, and it is often desirable to process chromatic and luminance signals separately. This means that colour detection techniques often involve separating the chromaticity signal from the luminance signal. In immunostain detection, it is the chromaticity signal or the colour spectrum that is of interest rather than absolute brightness. The chromaticity signals encode the spectral information of the stain and can therefore be used to detect positive staining. From a computational perspective using a 2D chromaticity space makes it easier to model the probability density function.

3 $$\begin{aligned} r=\frac{R}{R+G+B}\quad g=\frac{G}{R+G+B}\quad b=\frac{B}{R+G+B}\end{aligned}$$

4 $$\begin{aligned} rg=r-g\quad by=\frac{r+g}{2}-b \end{aligned}$$

5 $$\begin{aligned} Cb=- 0.1687\times R - 0.3313\times G + 0.5\times B \end{aligned}$$

6 $$\begin{aligned} Cr=0.5\times R - 0.4187\times G - 0.0813\times B \end{aligned}$$

7 $$\begin{aligned} Prob((rg,by)|S)=\frac{\#S[(rg,by)]}{N_{S}} \quad Prob((rg,by)|\overline{S})=\frac{\#\overline{S}[(rg,by)]}{N_{\overline{S}}} \end{aligned}$$

8 $$\begin{aligned} Prob((Cb,Cr)|S)=\frac{\#S[(Cb,Cr)]}{N_{S}} \quad Prob((Cb,Cr)|\overline{S})=\frac{\#\overline{S}[(Cb,Cr)]}{N_{\overline{S}}} \end{aligned}$$

In order to demonstrate that luminance is less important than chromaticity in stain colour detection we tested four colour models, in experiment step one, only some of which used luminance information. The models tested were the *RGB* colour model, the opponent colour model, the *YCbCr* model and the *CbCr* model. In the opponent colour model red–green (*rg*) and blue–yellow (*by*) chromaticity signals are derived from the original *RGB* input (Eq. 3). The Cb and Cr chromaticity signals are derived from the original RGB space (Eq. 5). Statistical colour models in the chromaticity space can then be constructed (Eqs. 7 and 8).

## Softwares

### Interactive tool in imageJ

We developed this colour detection method into a semi-automatic plugin in ImageJ which could be used to assist with IHC image analysis. The colour detection function is based on the statistical model presented in "Methods" section ; this allows rapid colour detection from arbitrary IHC stained slides. This tool was first published in [5], and here we modified the performance and added built-in models for the detection of stain colour in DAB and PSR stained specimens.

### Overview of application software

**Fig. 2:**
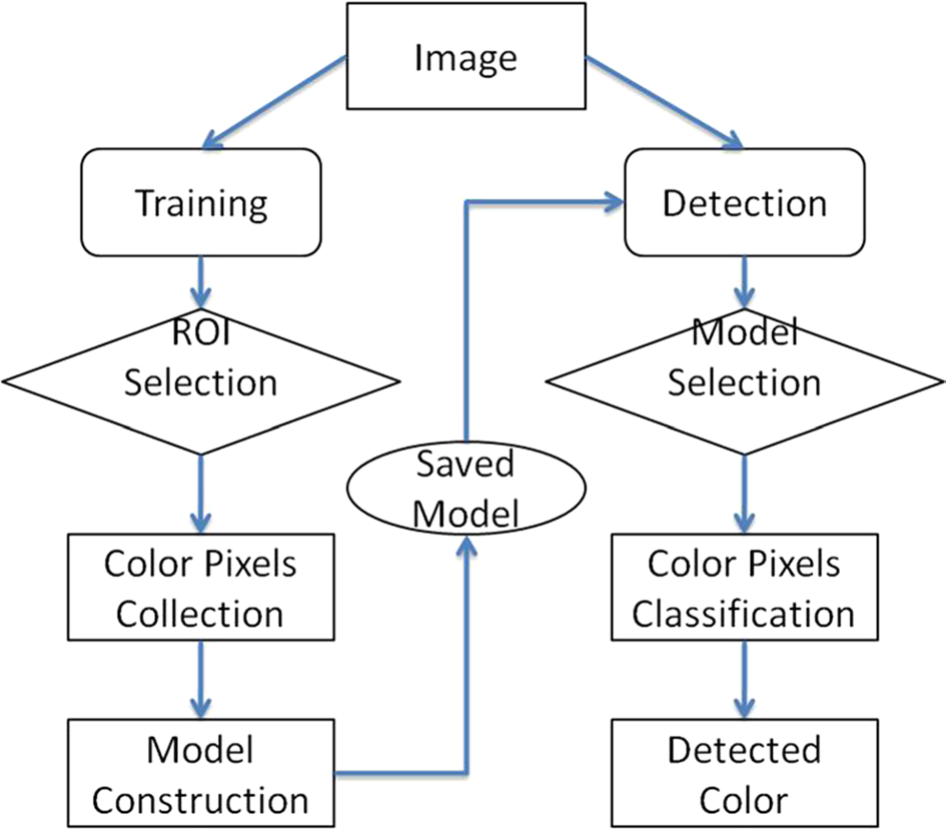
Workflow of stain colour detection in the interactive tool

The workflow for the interactive tool is presented in Fig. 2. We simplified the workflow in [5] to make it clear. It consists of two phases, the training and detection.

#### Training phase

**Fig. 3:**
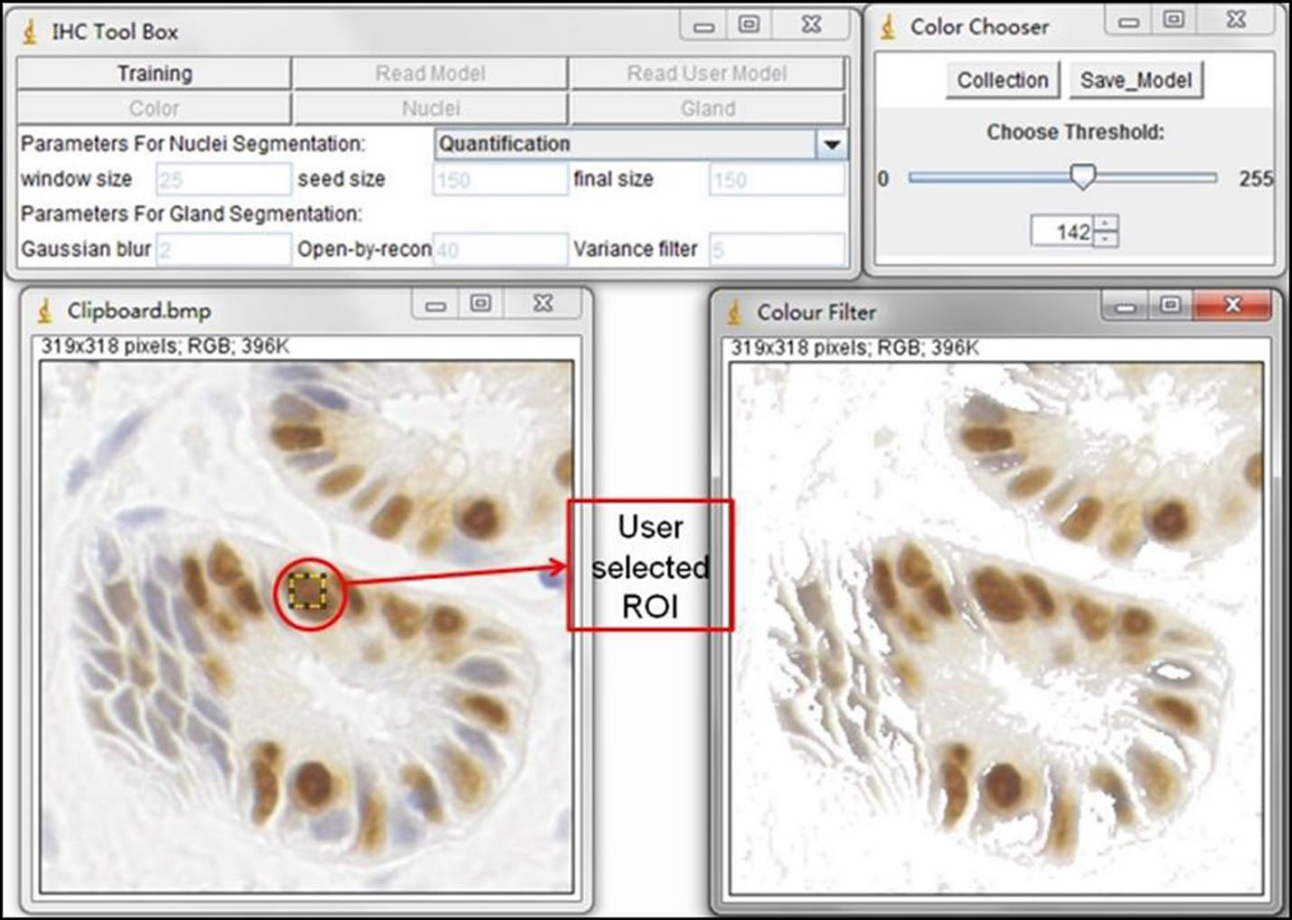
Stain colour detection in toolbox. This is an illustration of the training phase of colour detection. The *left image* is the original image. The *right image* is the output image. The colour pixels in the output image are colours similar to the colours in the selected ROI in the original image. The background pixels are removed by using the *scrolling bar*, and are set to be 255 in the output image

Users begin training by selecting a interested colour region (ICR) using a rectangular tool in ImageJ. There are two further components to this visual selection process; selection of the colour of interest and placement of a sliding bar within the scrolling panel, shown in Fig. 3. Background pixels can be filtered out using the sliding bar and appear as 255 in the resulting image. A statistical model is constructed from the histogram of the remaining colour pixels, which are quantified and collected. The training phase involves re-selecting an ICR in multiple training samples to obtain a wide range of shades of the target colour. When a new training sample is added the model is re-calculated automatically on the basis of the accumulated histograms.

#### Detection phase

When sufficient training samples have been collected, the statistical model created can be saved for reuse in subsequent detection phases. In the detection phase, the tool allows the user to use either the default DAB detection model obtained in our experiment, or a saved user-generated. The selected model can be used to detect similarly coloured stain in IHC images automatically.

## Experiments and discussion

### Data and three steps test

We proposed three steps experiments to evaluate statistical model and model based tool. Experiments were carried out in three sorts of dataset published in [5, 6]. We will publish these datasets with prepared ground truth online. The purpose of evaluation is comparing the computer model with human evaluation. However, there is no linked patient information for each image. We regarded the quantification of stain as quantification of colour. Thus, the evaluation was using randomly selected partial of images, in a dataset, to train model and using the rest of the images for testing. The training images were different from the test images.

**Table 1:** Number of patients of each datset and the number of images were captured and used for training and testing

Patients	Total images	Training images	Test images
WSI of oesophageal cancer			
74	60	10	50
TMA of colorectal cancer			
700	60	10	50
PSR stainlivercirrhosis			
15	25/60	5	20
DAB stainivercirrhosis			
100	48/189	10	38

**Fig. 4:**
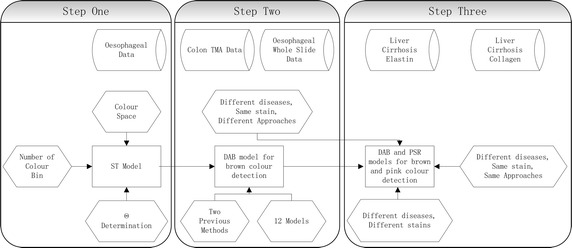
Flowchart of three steps of statistical model test

One sort of dataset was obtained from human oesophageal biopsy, one was obtained from human colorectal biopsy, and the other one was obtained from human liver cirrhosis biopsy. Slides come from hundreds of specimen and were prepared in different time and scanned using a Hamamatsu scanner. All slides were prepared by NHS Trust Nottingham University Hospital. Each whole slide (WS) comes from individual patient and tissue micro array (TMA) slides come from a total of 700 patients [13]. We show the number of images was used for training and testing in Table 1. These images were used in step two and step three. Whole Slide Images and TMA images, were randomly captured from oesophageal cancer dataset and colorectal cancer dataset, respectively. For PSR stained dataset, we separated 15 slides into 60 images with litter overlap regions and randomly selected 25 images to compromise the experimental dataset. And for DAB stained liver cirrhosis dataset, we separated 100 slides into 189 images with litter overlap regions and randomly selected 48 images to compromise the experimental dataset. The flowchart of carrying out experiments was shown in Fig. 4.

Images used in the first step, were captured from whole slides with DAB staining. We used this dataset to select the colour space, and determine paramenters, such as number of colour bins and $\theta$. This was already done in [6] and we specified the dataset and briefly described the experimental results in this paper. In step two, slides were prepared with two approaches, WS and TMA. Both of them were stained by DAB staining. In this step, we assessed user-independence and detection accuracy as comparing with two previous methods [5]. Datasets used in step three were stained by PSR staining and DAB staining. This step assessed the proposed model in detecting of different stain colour [5] and the same stain used on another disease [7]. In this paper, we added tests, in step two and step three, to evaluate the variations of detection results among vectors and statistical models. We also added tests to evaluate statistical model in histochemical stain detection and compared it with CD method. Each dataset was prepared in different magnification and resolution. We explained them one by one in the following three steps.

### Step one: testing the tool using different colour models

#### Dataset

The data for building the statistical colour models included 20 images with a resolution of 6720 × 4200. The models were then tested on another set of 75 images with the same resolution. Both the training and testing images were captured under 20× magnification. They were randomly captured from 14 whole slides.

#### Experiment

This semi-automatic tool was first used to label colour-positive pixels manually as described in "Softwares" section from 20 training images. Labeled pixels were collected and quantized into histogram bins to construct the statistical model based on (Eqs. 1, 7 and 8). The ground truth of the test dataset was also prepared manually by using this tool to eliminate all negative colour pixels.

The tested colour spaces were listed in "Methods" section. Two spaces which only use chromaticity channels are included: the opponent colour space and CbCr space. It is interesting to note that the CbCr chromaticity space has the smallest number of overlapping bins and the experimental results confirmed that this space gave the best performance.

This indicates that chromaticity is sufficient for accurate colour representation and that luminance is a distraction when building the model. As mentioned before relying on 2D chromaticity signals makes the model simpler, faster to compute and less demanding of memory. The optimal number of histogram bins is 128; this number produced better results than 256-bin histograms at a smaller computation cost. Please see more details in [6, 7].

### Step two: user-independence of the model

#### Dataset

Images were randomly captured from 74 whole slides and 14 TMA slides. Each TMA slide contained 16 × 7 cores. We randomly captured 60 images from either kinds of slide. The training dataset contained 10 WS images with a resolution of 6720 × 4200 and 10 TMA images with a resolution of 5120 × 4096. Both kinds of images were re-sorted into three sets of training samples. Each set of training samples consisted of 10 images, such as 10 WS images, 10 TMA images or a mixed set of 10 images (5 WS images and 5 TMA images). The test datasets were two datasets comprising DAB-stained WS images or DAB-stained TMA images. Both test datasets consisted of 50 images captured under 40× magnitude with a resolution of 1680 × 1050.

#### Experiment

Since the construction of the statistical model is based on collecting colour pixels using an interactive tool, models constructed by different users may produce different detection results when applied to a given set of images. It was therefore important to evaluate the robustness of the tool-generated statistical colour detection models. The robustness of statistical colour models created with the interactive tool was evaluated by measuring detection accuracy and variations in detection.

Four users participated in an experiment investigating detection of the brown colour in DAB-stained IHC images. All four users used the same training dataset to create models using the interactive tool. These models were then tested on the same test datasets, which were different from the training sets. As users may differ in what colours they classify as ’brown’. We calculated their true-positive ratio and false-positive ratio separately.

Each user was required to build three statistical models to detect brown colour. The colour pixels used were collected separately from each set of DAB-stained training samples. In this way the four users created 12 models that were automatically generated from the collections of colour pixels they selected using the interactive tool.

Models based on TMA training images were tested by TMA test images and models constructed by WS training images were tested by WS test images. The models created by mixed set of images were tested by both test images. These mentionded tests and results have been shown in [5, 7]. Here, we added tests to assess the variations of model-transfer, such as the models constructed by WS training images were tested by TMA test images and the models constructed by TMA training images were tested by WS test images.

**Fig. 5:**
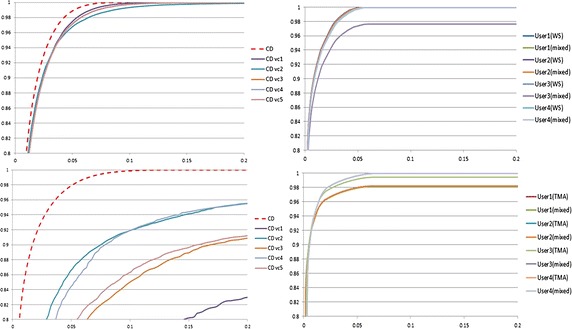
ROC *curves* for different user constructed statistical models. The plot at the *top left* is ROC curves for user obtained 5 vectors (based on 5 WS images) on 50 WS test images; The plot at the *bottom left* is ROC curves for 5 vectors (based on 5 TMA images) on 50 TMA test images; The plot at the *top right* is ROC curves for user constructed statistical models (based on 10 WS images and 10 mixed images) on 50 WS test images; The plot at the *bottom right* is ROC curves for user constructed statistical models (based on 10 TMA images and 10 mixed images) on 50 TMA test images. The *horizontal axis* is false-positive ratio and *vertical axis* is true-positive ratio

The results obtained are presented as receiver operating characteristic (ROC) curves with a true positive ratio (*TPR*) and false positive ratio (*FPR*) in Fig. 5. These ratios were calculated from (Eq. 9)

9 $$\begin{aligned} TPR=\left(\sum _{j=1}^{i} \frac{T_j}{G_S}\right)\arrowvert {i \in (1 \sim 200)} \quad FPR= \left(\sum _{j=1}^{i} \frac{D_j-T_j}{G_{\overline{S}}}\right)\arrowvert {i \in (1 \sim 200)} \end{aligned}$$

where $T_j$ is the truely detected number of stained positive pixels at intensity level *j*, $G_S$ is the total number of stained positive pixles in ground truth, $D_j$ is the totally detected number of pixels at intensity level *j* and $G_{\overline{S}}$ is the total number of stained negative pixels in ground truth. The true-positive and false-positive ratios were cumulatively calculated in the histogram bins from $i \in (1 \sim 200)$. All 12 models yielded good results, having high true-positive ratios and low false-positive ratios. These results showed there were small variations between models generated by different users in terms of the true-positive and false-positive ratios. For example, for the tests based on WS test images, at a threshold of 200 ($i = 200$ at Eq. 9), the four user-generated models, either trained by WS or TMA training images, all had the true-positive ratio close to 100 % but the false-positive ratio varied between 7.8 and 8.8 %; for the tests based on TMA test images, they had the true-positive ratio varied between 98.0 and 100 % and the false-positive ratio varied between 14.1 and 15.9 %.

### Step two: comparison of the statistical colour detection method with other methods

#### Dataset

The dataset used in the comparative study was the same as that used in the robustness evaluation reported in previous experiment.

#### Experiment

In this study we compared the statistical method with two previously developed colour detection methods in widespread use [9, 10]. All the methods were trained and tested based on the same datasets, which were prepared from different types of images of IHC staining. We compared the terms of detection accuracy, separation of stain colours, and variations between user trained models and vectors.

##### Accuracy of colour detection in DAB-stained samples

**Table 2:** 10 CD vectors obtained from 5 WS training images and 5 TMA training images for brown colour detection

Images	Whole slide			TMA		
	R	G	B	R	G	B
1	0.44950655	0.60628647	0.65601873	0.5197582	0.60258	0.6055978
2	0.44893575	0.6022728	0.6600941	0.52838445	0.5891207	0.6113483
3	0.40408775	0.6007869	0.6897595	0.55297637	0.5856209	0.5926764
4	0.39064342	0.5893953	0.70711446	0.4932045	0.59396774	0.6355719
5	0.37070048	0.5971876	0.7113003	0.5172909	0.59305906	0.6170017

The average results from four users using the new method were compared with results obtained from colour deconvolution (CD) and CMYK models. The study was conducted in ImageJ. Colour deconvolution was programmed by Landini as a plugin for ImageJ [14] based on the National Institutes of Health Image macro. Instead of using single DAB-stained sample, we trained the CD vectors by the same 10 training samples, 5 WS images and 5 TMA images in mixed set, as used in previous experiment. Vectors for brown colour of DAB stain were obtained through ImageJ Plugin. Each vector was obatined from one training image. They were tested by the corresponding test images. The results were shown in Table 2. The results with the 10 vectors obtained in this way were much worse than those obtained with the built-in H-DAB vector ($R = 0.26814753, G = 0.57031375, B = 0.77642715$) (see Fig. 5); we therefore used the built-in vector to discriminate the brown colour from the background in the evaluation. The CMYK model was also developed in ImageJ based on the functions mentioned in [15].

**Fig. 6:**
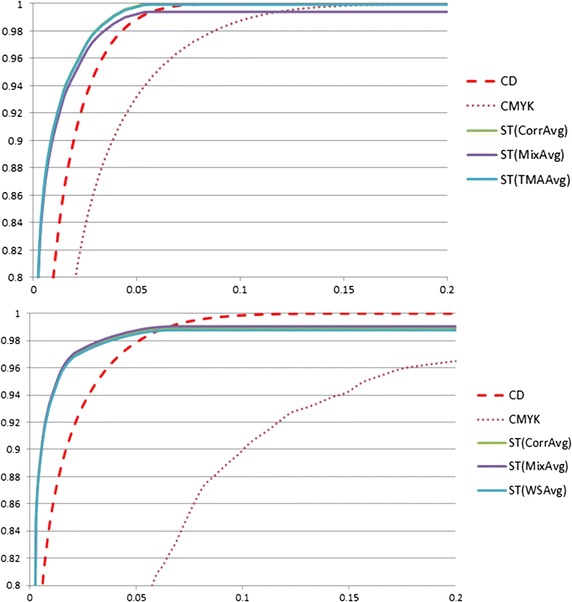
ROC curves of CD, CMYK, mean value of ST with three sets of training samples. Colour Deconvolution *CD*, CMYK colour space *CMYK*, mean value of ST models trained from corresponding images *CorrAvg*, mean value of ST models trained from mixed training images *MixAvg*, mean value of ST models trained from WS (TMA) training images *WS(TMA)Avg*. The plot at the *top* shows the ROC curves for CD (*red dashed line*), CMYK (*brown dotted line*), the mean value of ST models with corresponding training images (*smooth green line*), the mean value of ST models with mixed training images (*smooth purper line*), and the mean value of ST models with TMA training images (*smooth blue line*) on 50 WS test images; the plot at the *bottom* shows the ROC curves for CD, CMYK and the mean value of ST models with three sets of traning images (WS, Mixed and TMA training images) on 50 TMA test images. The *horizontal axis* is false-positive ratio and *vertical axis* is true-positive ratio

As shown in Fig. 6, both in WS and TMA datasets, almost methods produced highly accurate results. Statistical models with corresponding training images and CD with built-in vector were achieving close to 100 percent true-positive rate. The mean values of the statistical models, achieved the best results over the 50 WS test images, with a false-positive rate of 8.3 %; the false-positive rate 12.0 % for the CD method. The CMYK model had the lowest rate, true-positive rate, 92.9 %. The statistical models also achieved the best result for the set of 50 TMA test images, a 14.9 % false-positive rate compared with 16.6 % for the CD method. Again the CMYK model had the lowest true-positive rate, 91.4 %.

**Table 3:** The AUC values of the ROC curves of 12 models, when false positive ratio equals 10  %

	Whole slide			TMA		
	Model (WS) (%)	Model (TMA) (%)	Model (mix) (%)	Model (WS) (%)	Model (TMA) (%)	Model (mix) (%)
User1	97.2	97.0	97.1	95.8	95.9	95.9
User2	97.2	97.1	97.1	95.8	95.9	95.9
User3	97.0	96.9	94.6	95.8	96.6	96.6
User4	96.9	96.9	96.9	96.6	96.6	96.6
AVG	*97.1*	*97.0*	*96.5*	*96.0*	*96.2*	*96.2*
*CD*		93.9			94.5	
*CMYK*		86.1			69.3	

*WS* whole slide test images; *TMA* TMA test images; *ST*(*WS*) statistical model constructed on 10 WS training images; *ST*(*TMA*) statistical model constructed on 10 TMA training images; *Mix* statistical model constructed on 10 mixed training images (5 *WS* images and 5 *TMA* images); *AVG* average values of four user-detected results; *CD* colour deconvolution

To clarify these results, we calculated AUROC (area under ROC curve). Table 3 shows that the statistical colour models produced the best results. CD produced much better results than CMYK on both WS and TMA test images. Table 2 also shows that the user-generated models had varied slightly in terms of detection accuracy. For example, for brown colour detection, the lowest AUC was 94.6 % and the maximum was 97.2 %.

These results indicate that models generated by different users using this tool are all highly accurate and therefore that the method is robust and fairly user-independent. However, CD method with trained vectors has obvious variations in detection results, especially in detection of DAB stained TMA images, see Fig. 5. The results also show the mixed models and models constructed not from corresponding training images can generate similar results to the models only trained by corresponding training images. It demostrates the model constructed by the whole range of colour shades can be adopted in different datasets obtained from different diseases for the same stain colour detection.

##### Dark stain colour detection

**Fig. 7:**
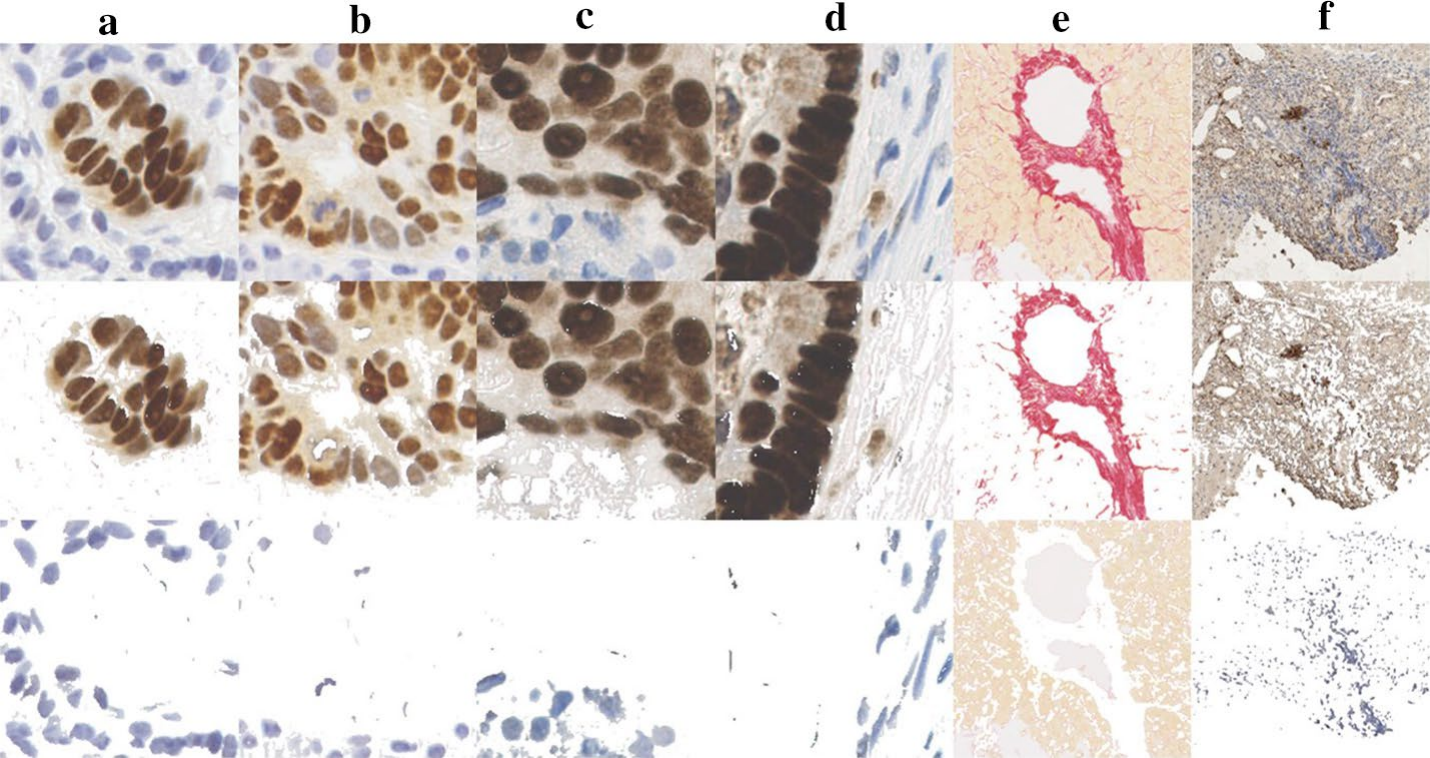
The statistical model detected stain colour on IHC stained samples. **a**, **b** columns are the DAB-stained samples from WS images; **c**, **d** columns are the DAB-stained samples from TMA images; **e** column is a PSR-stained liver cirrhosis sample; **f** column is a DAB-stained liver cirrhosis sample showing elastin fibrosis. The images in *top row* are original IHC stained images; the images in *middle row* are model-detected *brown* or *pink colour*; the *bottom row* is model-detected counter stain colour, *blue* or *yellow*

Normal brown colour was detected easily and separated from the background by all three methods. Detection of brown coloration in a dark-stained slide is more challenging however; the CMYK method undercounted dark brown-coloured pixels (Fig. 1d, e) whereas CD falsely detected dark brown as the colour blue (Fig. 1b, c). This evaluation of CMYK demonstrated that a colour space-based method performed less accurately in stain colour detection [16]. Classification of multi-stain colours in colour space may be affected by overlap. The CD method also suffers from this problem and the non-linear light absorption of DAB stain. The statistically-based interactive tool detected dark brown and blue correctly (see Fig. 7).

### Step three: use of statistical colour models in assessment of human histopathology

#### Dataset

We prepared two datasets in this experiment, one contained 25 images randomly selected from 60 images stained with PSR, and the other contained 48 images randomly selected from 189 slides stained with DAB. In the former dataset, we created a statistical colour model using a training set of 5 PSR WS images with a resolution of 3360 × 2100. The test dataset consisted of 20 PSR-stained images, captured under 5× magnification with a resolution of 3360 × 2100. For the later, the training samples used to create the model were 10 images with a resolution of 5600 × 4200. The model was then tested on a large dataset consisting of 38 images, captured under 5 ×  magnification with a resolution of 5600 × 4200.

#### Experiment

The statistical colour model can be adapted to detect pixels of any colour, not just the brown target pixels typical of IHC. The PSR stain is used to assess fibrosis in liver tissue; it stains the connective tissue matrix pink and background liver tissue pale yellow.

Elastin accumulates in the liver as fibrosis progresses [17] and can be specifically detected using IHC. The target pixels are stained brown with blue counter-stain. We applied the statistical detection method to the detection of brown colour in liver cirrhosis biopsies stained for elastin fibres.

The study was conducted in ImageJ. Five vectors used for PSR detection using CD were obtained from 5 training images. The best of the five vectors with the highest true positive ratio and lowest false positive ratio was used for pink colour detection evaluation comparison(vectors for pink colour of PSR, R = 0.12670784, G = 0.76997238, B = 0.62432366). We trained another five vectors of CD on 5 of 10 elastin training images. We used the best of five vectors and built-in vector for brown colour detection evaluation comparison.

**Fig. 8:**
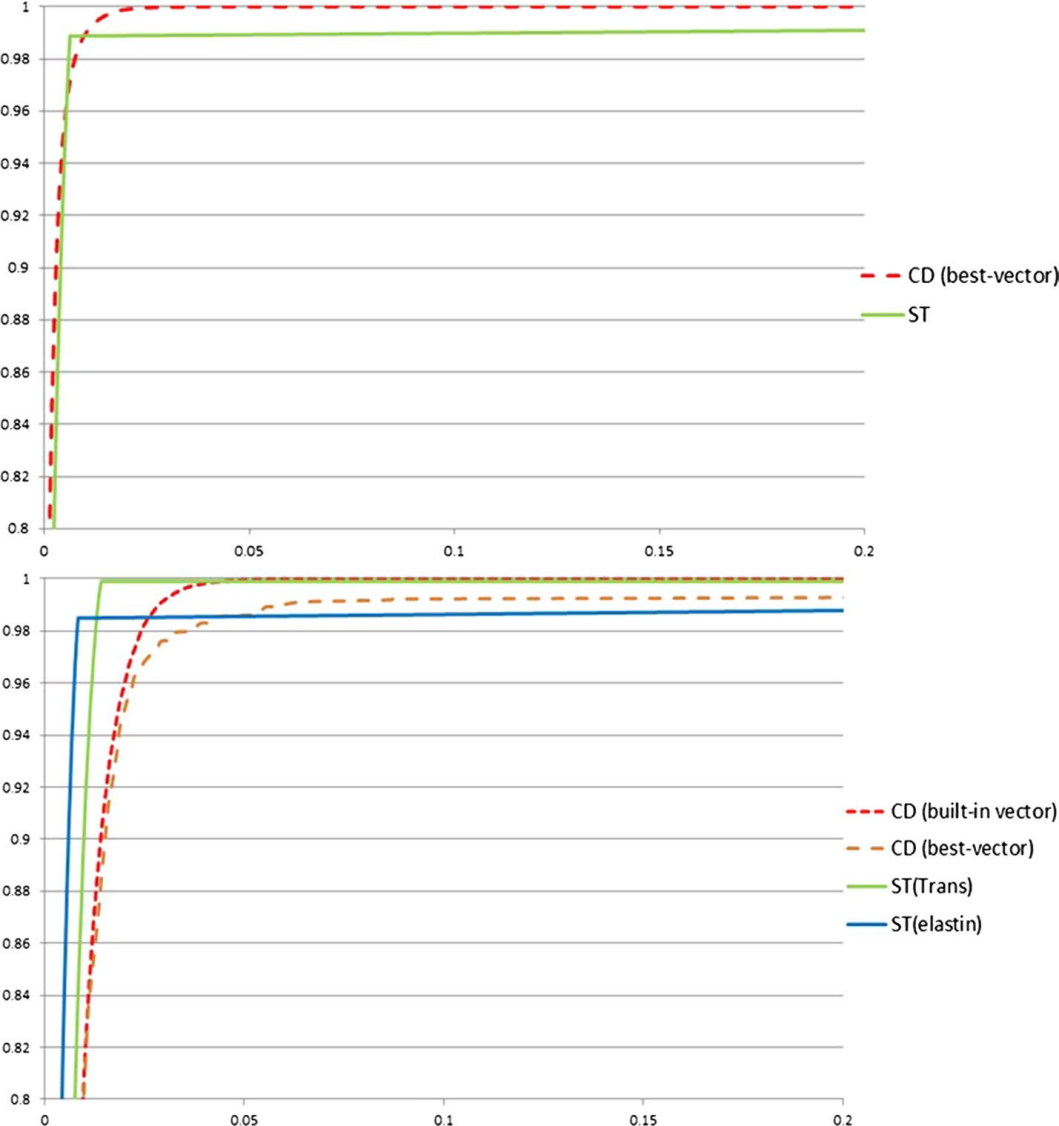
ROC curves for CD and ST model for the PSR and elastin datasets. Plot at the *top* is the ROC for *pink colour* detection on PSR stained images, the ROC curve for CD with best-vector (CD(best-vector)) is *red dashed line*, the ROC curve for ST model with PSR stained training images (ST) is *smooth green line*; The plot at the *bottom* is the ROC for *brown colour* detection on DAB stained elastin images, The ROC curve for CD with built-in vector (CD(built-in)) is *red dashed line*, the ROC curve for CD with best-vector (CD(best-vector)) is *orange dashed line*, the ROC curve for ST transferred model (ST(trans)) is *smooth green line*, and the ROC curve for ST model with DAB stained elsatin training images (ST(elastin)) is *smooth blue line*. The *horizontal axis* is false-positive ratio and *vertical axis* is true-positive ratio

**Table 4:** The AUC values of the ROC curves of pink colour detection in PSR stained images and brown colour detection in DAB stained elastin images, when false positive ratio equals 10 %

	PSR			
	ST model (%)		CD best vector (%)	
TPR	98.9		100	
FPR	0.6		9.1	
AUC	97.7		99.0	

	DAB–elastin			
	Model (ST trans) (%)	Model (ST elastin) (%)	Model (CD builtin) (%)	Model (CD best vector) (%)
TPR	99.9	98.5	100	99.2
FPR	1.4	0.8	9.3	8.8
AUC	95.1	96.0	93.9	92.6

*STtrans* a model of 12 models which achieved the best performance; *STelastin* model constructed from 10 DAB stained elastin training samples; *CDbuiltin* colour deconvolution; *CD best vector* a vector of 5 vectors which achieved the best performance; *TPR* true positive ratio; *FPR* false positive ratio

The ROC curves for pink colour detection and brown colour detection are shown in Fig. 8. The specified resutls are shown in Table 4. For pink colour detection, statistical model and CD method obtained similar results. In particular, statisical model had fewer true-positive rate and AUC value than CD, but a 0.6 % false-positive rate compared with 9.1 % for the CD method.

For the detection of brown colour, we used two statistical models mentioned above. Although both models and CD method achieved highly accurate results, the statistical models achieved much better results, both in accuracy and AUC. The transferred statistical model in results was simlar to the model trained from corresponding training images. It also demostrates the model constructed by the whole range of colour shades can reduce the inter-dataset variation.

We compared the methods in calculating the percentage of pink-coloured pixels or brown-coloured pixels represented in the slide, and the correlation between the detected results and the manually calculated results [5]. The calculation process was similar to [8]. The manually calculated results were obtained by using the manually labelled stain colour against the tissue slide. Results have shown statistical model can achieve higher $R^2$ than CD method in detecting of DAB stain and have equal $R^2$ with CD method in detection of PSR stain.(PSR: ST 0.9994 vs CD 0.9823; DAB: ST 0.8658 vs CD 0.5183).

## Conclusions

It is clear that stain colour detection is similar to normal colour detection in computer vision. A statistical model can also produce good results in medical image analysis. The statistical model combined with an interactive human training process yielded better results than CD or CMYK methods with the DAB-stained tissue samples. This study has demonstrated that the tool we have developed, which is based on a statistical model, to colour detection is in concordance with human evaluation.

The accuracy of the model may be affected by the colour space selected and the collected colour pixels used to train the model. Four colour spaces were compared in the DAB colour detection study, which compared detection accuracy using the different chromomeric channel domains. RGB colour space and the absolute luminance channel can be discarded to reduce computation costs and reduce the requirement for computer memory.

The model is generated from colour pixels collected from a set of training images using an interactive tool. Although the tool makes the colour pixel collection process easier, individual human differences might affect detection accuracy, so we evaluated the robustness of the method, by testing 12 models generated by four different users from three sets of training images on the same test dataset. Detection accuracy varied only slightly between users and there were no obvious inter-observer differences.

It is commonly that slides prepared with different approaches or from different diseases may stain with same colour. Models constrcuted from one dataset might not be transferred to another dataset for the same stain colour detection with same accuracy. We considered this issue and crossly tested trained models among different datasets. Results have shown only slightly variations between these models and there were no obvious inter-dataset performance degradation.

Model detects IHC and histochemical stain colour stable and efficient. The tool makes the model customization very easy. The user should aim to collect pixels representing the whole range of colour shades. Collecting a sufficiently large sample of colour pixels may reduce both inter-observer and inter-dataset differences.
